# Supplementary material for: Conjunction of factors triggering waves of seasonal influenza
Source: eLife. 2018 Feb 27;7:e30756. doi: 10.7554/eLife.30756 (PMC5864297; doi:10.7554/eLife.30756)
Supplement: Supplementary file 1. — SI2.docx [file elife-30756-supp1.docx]

**1**

**Summarized Algorithms for Non-parametric Granger Causal Inference**

**Algorithm 1:** Stream-run Function

**Input**: Strongly connected labeled graph G = (Q, Σ, δ), string s *∈* Σ***

**Output**: ρ(G, s)

**1** Initialize zero vector v of length |Q|

**2** Choose random node qk** ∈* Q

**3** qcurrent *←* qk***

**4** vk** ←* 1

**5 for** i *←* 1 *to* |s| **do**

**6** qcurrent *←* δ(qcurrent, si)

**7 if** qcurrent == qk **then**

**8** vk *←* vk + 1

**9 for** k *←* 1 *to* |Q| **do**

**10** vk *←* vk/ )j vj

# /* Normalize vector */

**11 return** ρ(G, s) *←* v

**Algorithm 2:** Efficient Computation of the Coefficient of Dependence

**Input**: £, si, sj

**Output**: Estimate γi for γi

j j

# // Compute XPFSA

**1** Compute Hi

j

// Compute φsj

λ

**2** r *←* [0 *· · ·* 0]

// Length = |Σj|

**3 for** k *←* 1 *to* |sj| **do**

**4 if** sj[k] == σ£ **then**

**5** r£ *←* r£ + 1

# // Compute denominator

|Σj|

**6** h0 *←* \ rk log2(rk)

k=1

# // Compute numerator

**7** h1 *←* 0

**8** u *←* ρ(Hi, si)

j

**for** *←* 1 *to* |Qi| **do**

**9**

k j

**10** h[k] *←* \

�j(qk, σ£) log2

(

�j(qk, σ£)

πi

σ *∈*Σj

πi

**11** h1 *←* h1 + ukh[k]

**12 return** γi *←* h1

j h0

**Algorithm 3:** Prediction of Next-symbol Distribution From Cross-talk

**Input**: £, si, sB, xi

**Output**: Predicted next-symbol distribution τi

# // Compute PFSA & XPFSA

πi i

**1** Compute Hi = (Qi, Σi, δi, � ) using s , £

B B B πi i B

**2** Compute Hi = (Qi , Σi, δi , �B) using s , s , £

**3** G *←* Hi *−→*Hi

*⊗* B

**4** Compute stationary distribution ℘G

λ

**5 foreach** σ *∈* Σi **do**

**6** Compute Γ G

σ

**7** ℘0 *←* ℘G

λ

**8 for** k *←* 1 *to* |xi| **do**

℘0Γ G

i

x

**9** ℘0 *←* k

℘0Γ G

i

x

k 1

**10 return** τi *←* ℘0Π� i

B
