# Supplementary material for: Conjunction of factors triggering waves of seasonal influenza
Source: eLife. 2018 Feb 27;7:e30756. doi: 10.7554/eLife.30756 (PMC5864297; doi:10.7554/eLife.30756)
Supplement: Supplementary file 2. [file elife-30756-supp2.docx]

**Supplementary File 2: Back to School Effect Analysis**

TABLE S1: Weeks considered as trigger periods, and match with school open times in US

# Running Fisher’s Exact Test

| Match | Start | End | **T**  *•* |
| --- | --- | --- | --- |
| 1 | 12-31-2002 | 01-06-2003 |  |
| 1 | 01-07-2003 | 01-13-2003 |  |
| 1 | 01-14-2003 | 01-20-2003 |  |
| 1 | 09-23-2003 | 09-29-2003 |  |
| 1 | 09-30-2003 | 10-06-2003 |  |
| 1 | 10-07-2003 | 10-13-2003 |  |
| 0 | 10-14-2003 | 10-20-2003 |  |
| 0 | 10-21-2003 | 10-27-2003 |  |
| 0 | 11-09-2004 | 11-15-2004 |  |
| 0 | 11-16-2004 | 11-22-2004 |  |
| 0 | 11-23-2004 | 11-29-2004 |  |
| 0 | 11-30-2004 | 12-06-2004 |  |
| 0 | 12-07-2004 | 12-13-2004 |  |
| 0 | 12-14-2004 | 12-20-2004 |  |
| 0 | 11-01-2005 | 11-07-2005 |  |
| 0 | 11-08-2005 | 11-14-2005 |  |
| 0 | 11-15-2005 | 11-21-2005 |  |
| 0 | 11-22-2005 | 11-28-2005 |  |
| 0 | 11-29-2005 | 12-05-2005 |  |
| 0 | 12-06-2005 | 12-12-2005 |  |
| 0 | 12-13-2005 | 12-19-2005 |  |
| 1 | 10-03-2006 | 10-09-2006 | *•* |
| 0 | 10-10-2006 | 10-16-2006 | *•* |
| 0 | 10-17-2006 | 10-23-2006 |  |
| 0 | 10-24-2006 | 10-30-2006 |  |
| 0 | 10-31-2006 | 11-06-2006 |  |
| 0 | 11-07-2006 | 11-13-2006 |  |
| 0 | 11-14-2006 | 11-20-2006 |  |
| 0 | 11-13-2007 | 11-19-2007 |  |
| 0 | 11-20-2007 | 11-26-2007 |  |
| 0 | 11-27-2007 | 12-03-2007 |  |
| 0 | 12-04-2007 | 12-10-2007 |  |
| 0 | 12-11-2007 | 12-17-2007 | *•* |
| 0 | 12-18-2007 | 12-24-2007 |  |
| 0 | 11-25-2008 | 12-01-2008 |  |
| 0 | 12-02-2008 | 12-08-2008 |  |
| 0 | 12-09-2008 | 12-15-2008 |  |
| 0 | 12-16-2008 | 12-22-2008 |  |
| 0 | 12-23-2008 | 12-29-2008 |  |
| 0 | 12-30-2008 | 01-05-2009 |  |
| 0 | 06-30-2009 | 07-06-2009 |  |
| 0 | 07-07-2009 | 07-13-2009 |  |
| 0 | 07-14-2009 | 07-20-2009 |  |
| 0 | 07-21-2009 | 07-27-2009 |  |
| 0 | 07-28-2009 | 08-03-2009 |  |
| 0 | 08-04-2009 | 08-10-2009 |  |
| 0 | 10-19-2010 | 10-25-2010 |  |
| 0 | 10-26-2010 | 11-01-2010 |  |
| 0 | 11-02-2010 | 11-08-2010 |  |
| 0 | 11-09-2010 | 11-15-2010 |  |
| 0 | 11-16-2010 | 11-22-2010 |  |
| 0 | 11-23-2010 | 11-29-2010 |  |

**o Determine School Opening Effect on Epidemic Trigger**

School-open influence assumed in:

-- 1 week in August,

-- 4 weeks in September,

-- 1 Week in October,

-- 2 weeks in January

(8 weeks total) Match found: 7 weeks Confusion matrix:

Result from Fisher’s exact test:

-- p-value = 0.8391

-- alternative hypothesis: true odds ratio is not equal to 1

-- odds ratio = 0.8403

|  | flu initiates no initiation |
| --- | --- |
| school-opening weeks otherwise | 7 65  45 351 |
