# Supplementary material for: Conjunction of factors triggering waves of seasonal influenza
Source: eLife. 2018 Feb 27;7:e30756. doi: 10.7554/eLife.30756 (PMC5864297; doi:10.7554/eLife.30756)
Supplement: Supplementary file 4. — Balancing Precision and Sensitivity when Identifying Influenza-like Illnesses from Electronic Medical Records. [file elife-30756-supp4.docx]

# Supplementary File 5: Balancing precision and sensitivity when identifying influenza-like illnesses

**from electronic medical records**

Viboud *et al.* [[1](#_bookmark0)] suggested the method of identifying influenza-like illness in electronic medical records, using the following logical combination of International Classification of Disease (version 9-CM): 11(ICD-9 487-488 OR [780.6 and (462 or 786.2)] OR 079.99).”

In following Viboud et al.’s recommendation, we found ourselves faced with two choices: (1) select a smaller number of patients that are most likely to have true ILIs using ICD-9-CM codes 487 and 488, or; (2) select a larger (more than double) number of patients including numerous non-related cases by also including codes such as 079.99.

By definition, ILIs are everything that looks like influenza to a physician: ICD-9-CM codes 487 and 488 cover all such cases.

- 487 Influenza;
- 487.0 Influenza with pneumonia;
- 487.1 Influenza with other respiratory manifestations;
- 487.8 Influenza with other manifestations;
- 488 Influenza due to certain identified influenza viruses;
- 488.0 Influenza due to identified avian influenza virus;
- 488.01 Influenza due to identified avian influenza virus with pneumonia;
- 488.02 Influenza due to identified avian influenza virus with other respiratory manifestations;
- 488.09 Influenza due to identified avian influenza virus with other manifestations;
- 488.1 Influenza due to identified 2009 H1N1 influenza virus;
- 488.11 Influenza due to identified 2009 H1N1 influenza virus with pneumonia;
- 488.12 Influenza due to identified 2009 H1N1 influenza virus with other respiratory manifestations;
- 488.19 Influenza due to identified 2009 H1N1 influenza virus with other manifestations;
- 488.8 Influenza due to novel influenza A;
- 488.81 Influenza due to identified novel influenza A virus with pneumonia;
- 488.82 Influenza due to identified novel influenza A virus with other respiratory manifestations;
- 488.89 Influenza due to identified novel influenza A virus with other manifestations.

Other code choices are very non-specific, consider “[780.6 and (462 or 786.2)]:”

- 780.6 Fever and other physiologic disturbances of temperature regulation;
- 462 Acute pharyngitis;

*•* 786.2 Cough.

Thus, a patient having fever and either cough or pharyngitis would be classified as having influenza. Code 079.99 (Unspecified viral infection) is even less specific: By definition, it covers all viral infections that cannot be assigned precisely by a physician and are not influenza-like. The following list of conditions possibly fall under this category:

- Acute retinal necrosis;
- Acute viral bronchiolitis;
- Acute viral disease;
- Acute viral laryngotracheitis;
- Acute viral otitis externa;
- Acute viral thyroiditis;
- Amantadine resistant virus present;
- Arthritis due to viral infection;
- Arthritis of hand due to viral infection;
- Arthritis of knee due to viral infection;
- Arthropathy associated with viral disease;
- Boid inclusion body disease;
- Cardiomyopathy due to viral infection;
- Cardiomyopathy, due to viral infection;
- Congenital viral disease;
- Congenital viral infection;
- Disease of possible viral origin;
- Encephalitis due to influenza-specific virus not identified;
- Maternal viral disease complicating pregnancy;
- Maternal viral disease in pregnancy;
- Neonatal viral infection of skin;
- Nonspecific syndrome suggestive of viral illness (finding);
- Oral mucosal viral disease;
- Postpartum (after childbirth) viral disease;
- Postpartum viral disease;
- Postviral depression;
- Postviral excessive daytime sleepiness;
- Postviral infection debility;
- Viral acute pancreatitis;
- Viral bronchitis;
- Viral carditis;
- Viral dermatitis of eyelid;
- Viral disease;
- Viral disease in childbirth;
- Viral disease in pregnancy;
- Viral ear infection;
- Viral esophagitis;
- Viral eye infection;
- Viral infection;
- Viral infection by site;
- Viral lower respiratory infection;
- Viral musculoskeletal infection;
- Viral myositis;
- Viral pleurisy;
- Viral respiratory infection;
- Viral retinitis;
- Viral syndrome;
- Viral ulcer of esophagus;
- Virus present;
- Zanamivir resistant virus present.

As our analysis is focused on influenza-like epidemic waves, it is less important for us to have a larger number of patients (almost certainly, most of them with non-influenza viral diseases) than to have a smaller number of patients who, most likely, were diagnosed with influenza

The addition of codes 079.99 to 487 as well as 488 does substantially increase the number of unique people with viral infection cases (over the whole period visible in the insurance claims) from 4,009,982 to 10,682,458 (more than twice the amount). However, according to code definitions provided above, this impressive increase has to come at the price of a substantial drop in precision (possibly 50 to 90 percent).

In the absence of a “gold standard” (a dataset where the virus from each patient was typed via sequencing) the best we can do is to rely on the intuition of the physician who recorded diagnosis in each case.

**R**eferences

[1] Viboud C, Charu V, Olson D, Ballesteros S, Gog J, Khan F, et al. Demonstrating the use of high-volume electronic medical claims data to monitor local and regional influenza activity in the US [Journal Article]. PLoS One. 2014;9(7):e102429. Available from: [http:*//*www.ncbi.nlm.nih.gov*/*pubmed*/*25072598](http://www.ncbi.nlm.nih.gov/pubmed/25072598).
